# Long-term outcome of patients with vaccine-induced immune thrombotic thrombocytopenia and cerebral venous sinus thrombosis

**DOI:** 10.1038/s41541-022-00491-z

**Published:** 2022-07-05

**Authors:** Sarah Kehr, Philipp Berg, Susanne Müller, Sarah A. Fiedler, Britta Meyer, Gabriele Ruppert-Seipp, Cornelia Witzenhausen, Marc E. Wolf, Hans H. Henkes, Doris Oberle, Brigitte Keller-Stanislawski, Markus B. Funk

**Affiliations:** 1 Paul-Ehrlich-Institut (Federal Institute for Vaccines and Biomedicines), Division Safety of Medicinal Products and Medical Devices, Langen, Germany; 2 Department of Neurology, Klinikum Stuttgart, Stuttgart, Germany; 3 Department of Neuroradiology Klinikum Stuttgart, Stuttgart, Germany

**Keywords:** Public health, Drug regulation

## Abstract

We present the long-term outcomes of 44 patients who developed cerebral venous sinus thrombosis after vaccination with the adenoviral vector ChAdOx1 nCoV-19 COVID-19 vaccine. Assessment of the Extended Glasgow Outcome Scale was performed within 3–6 months after the initial hospital admissions. Patient outcomes ranged from good recovery (13 patients, 29.6%) to moderate disability (11 patients, 25.0%) and severe disability or vegetative state (6 patients, 13.6%). Fatal outcomes were reported in 14 patients (31.8%).

## Introduction

Widespread vaccination will be critical to control the Severe Acute Respiratory Syndrome—Coronavirus (SARS-CoV)-2 pandemic. Several vaccines have already been approved by the European Medicines Agency (EMA). Despite a large number of clinical trial participants, the information available on vaccine safety is still limited during marketing authorization. Consequently, pharmacovigilance and close monitoring of all newly approved vaccines remain vital to identify previously unrecognized safety issues to be included in the ongoing risk-benefit analysis. In the spring of 2021, unanticipated thromboses, including cerebral venous sinus thrombosis (CVST) with thrombocytopenia, emerged as an adverse reaction (ADR) in patients who had been vaccinated with the ChAdOx1 nCoV-19 vaccine (Vaxzevria^®^), while this was not observed for the mRNA vaccines.

This adverse reaction was termed vaccine-induced immune thrombotic thrombocytopenia (VITT)^1–3^ or thrombosis with thrombocytopenia syndrome (TTS)^4^. Although sporadic in nature, VITT can result in severe disease in the individual vaccinee. A diagnosis of VITT with CVST can be life-threatening or result in long-term disability as CVST in this context is frequently associated with secondary intracranial hemorrhage and high mortality^5^. While data focusing on the acute clinical development of this condition have been presented^6,7^, very few findings are available that address clinical outcomes and the course of recovery from CVST with VITT. Therefore, we performed follow-up assessments of a series of patients who developed CVST with VITT in response to vaccination with Vaxzevria^®^ that were reported to the Paul-Ehrlich-Institut (PEI, German Federal Authority for Vaccines). Case reports were assessed regarding the outcome of the ADRs in vaccinees to evaluate their severity and late complications.

According to German law, suspected ADRs following immunization must be reported to the competent authorities. We followed up on the outcomes and status of neurological recovery of 49 cases of VITT with CVST that were reported to PEI in early 2021. Suspected diagnoses and initial ADR reporting can be initiated by medical facilities that are different from the primary specialty department for this newly emerging ADR. Likewise, therapy may continue in a separate rehabilitation facility. These factors can complicate follow-up and outcomes evaluation. For most patients, follow-up was pursued about 3–6 months after hospital admission. The reported outcomes describe the patients’ neurological status at 5–38 weeks (mean 20 weeks) after hospital admission. In two further cases, the neurological outcome was only available at discharge from patient treatment at the hospital and could not be further followed. Outcomes were identified in 44 of the original 49 cases. No information on clinical outcomes could be obtained from the remaining five patients (Suppl. Figure 1).

## Characteristics of the 49 patients with CVST

Forty-nine patient cases were evaluated in this investigation. Of these, 38 patients were female and 11 were male, which likely reflects vaccination recommendations at that time (please refer to the Supplementary Information for details) (Suppl. Table 1). The mean age was 45 years (range 19–73 years), and most patients (*n* = 34) were admitted to the hospital during week 2 after vaccination. Seven patients were admitted to the hospital during week 1 after vaccination and 8 patients were hospitalized during week 3 upon vaccination (overall mean 11 days, range 5–19 days). Spontaneous reporting details on ADR management are not provided on a routine basis, but medical reports were requested for this particular reaction. Twenty-three of these patients underwent a surgical procedure, including 15 patients who required craniectomies to reduce intracranial pressure (6 survived). Catheter-assisted thrombectomy was performed in 9 cases (including 2 craniotomies with thrombectomy). One patient required implantation of a ventricular drainage device due to cerebrospinal fluid circulation disorder. For 22 patients intravenous immune globulin (IVIG) treatment was reported. Half of these patients (*n* = 11) additionally underwent a surgical procedure. In 35 patients, treatment with low-molecular-weight heparin, non-heparin anticoagulants, or other medications such as corticosteroids and/or platelet transfusions was reported. In this context, it has to be noted that at the time when most reports were received, no guidelines for VITT treatment were available or they were about to be generated^8,9^. Concerning possible risk factors associated with CVST (Suppl. Table 2), two of the patients were smokers (smoking status was reported for 32 patients), and 9 patients used hormonal contraceptives (information on contraceptive intake was available for 24/38 female patients). Weight and height were known for 27 patients. However, only two patients presented with an abnormally high BMI >35 kg/m^2^. History of thrombosis was described in 1 patient, and only 1 patient showed two or more risk factors.

## Glasgow Outcome Scale

Clinical outcome data based on the Extended Glasgow Outcome Scale (GOS-E) were available for 44 of the 49 patients (Table 1). GOS-E grades ranged from good recovery in 13 patients (29.6%), moderate disability in 11 patients (25.0%), and severe disability/and vegetative state in 6 patients (13.6%). Fatal outcomes were reported in 14 patients (31.8%), all of these patients died during hospitalization and none during rehabilitation.

**Table 1:** GCS and GOS-E of patients with CVST.

Glasgow Coma Scale (GCS) at hospital admission			Glasgow Outcome Scale-Extended (GOS-E) 3–6 months after hospital discharge		
GCS Category	GCS Classification	Patients with reported data	GOS-E Category	GOS-E classification	Patients with the reported outcome
15	No disturbance of consciousness	15 (35.7%)	8	“Good recovery upper”	9 (20.5%)
14			7	“Good recovery lower”	4 (9.1%)
13	Minor disturbance of consciousness	10 (23.8%)	6	“Moderate disability upper“	5 (11.4%)
12			5	“Moderate disability lower”	6 (13.6%)
11	Significant disturbance of consciousness	6 (14.3%)	4	“Severe disability upper”	3 (6.8%)
10			3	“Severe disability lower”	2 (4.6%)
9			2	“Persistent vegetative state”	1 (2.3%)
≤8	Severe disturbance of consciousness	11 (26.2%)	1	“Death”	14 (31.8%)
Total		42 (100%)	Total		44 (100%)

Among the 14 patients with fatal outcomes (GOS-E score 1), the mean age was 50 years (range 25–73 years). Patients with severe disability or who remained in a persistent vegetative state (GOS-E scores 2–4) had a mean age of 49 years (range 20–64 years). The remaining patients (mean age 40 years; range 19–68 years) had either moderate neurological disabilities or good recoveries (GOS-E scores 5–8).

**Fig. 1:**
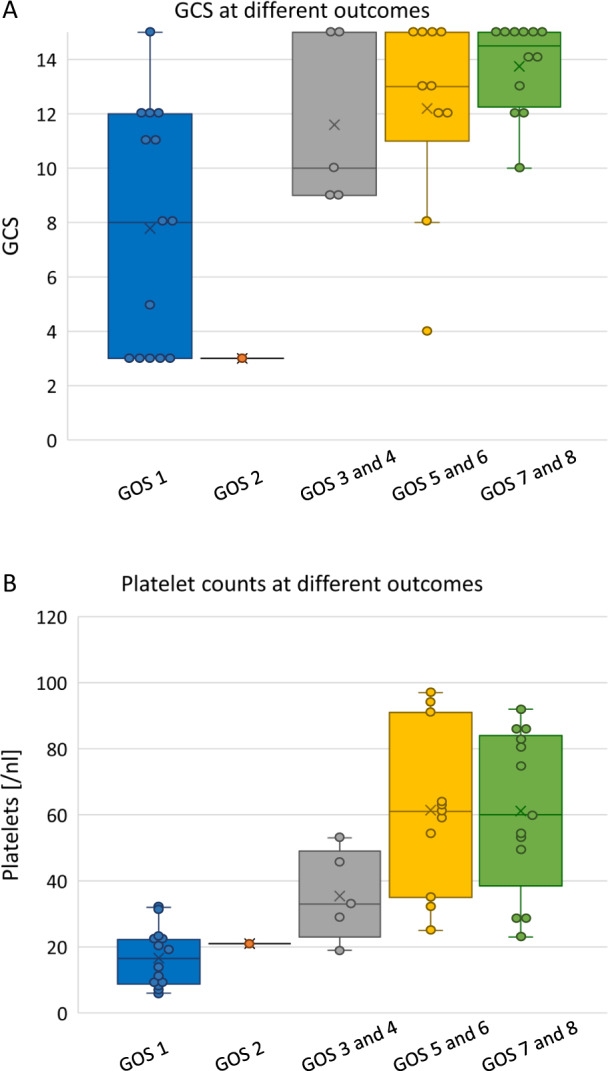
GCS and platelet counts at different outcomes. **A** GCS versus GOS of 42 patients with CVST. Patients with no information on GCS available are excluded (*n* = 42). Box plots show median (center line), mean (cross), 25% (Q1), and 75% (Q3) percentiles (interquartile range (IQR)), outliers, lower whisker as Q1–1.5 × IQR and upper whisker as Q3 + 1.5 × IQR. Each dot represents a single patient. Spearman Correlation Coefficient was calculated with *α* = 0.05 (*r* = 0.60, *P* < 0.001). **B** Outcome of 44 patients with CVST in relation to platelet count nadirs. Box plots show median (center line), mean (cross), 25% (Q1), and 75% (Q3) percentiles (interquartile range (IQR)), lower whisker as Q1–1.5 × IQR, and upper whisker as Q3 + 1.5 × IQR. Each dot represents a single patient. Spearman Correlation Coefficient was calculated with *α* = 0.05 (*r* = 0.73, *P* < 0.001).

To analyze the clinical outcomes with respect to patient state at the time of admission, we compared the Glasgow Outcome Scale (GOS) and the GCS scores available for 42 patients (Fig. 1A). GCS was determined for 42 patients with sufficient data (Table 1). For two of the patients, the neurological outcome (GOS-E 5 and 8, respectively) was only available at discharge from patient treatment at the hospital. These scores were divided into the following groups: GCS score 14–15 (15 patients, 36%), GCS score 12–13 (10 patients, 24%), GCS score 9–11 (6 patients, 14%), and GCS score ≤8 (11 patients, 26%).

The overall outcome and GCS at hospital admission were correlated (Spearman correlation coefficient, *r* = 0.60, *p* < 0.001) and were better in individuals who presented with no or only minor disturbances of consciousness at the time of admission (GCS scores 12–15) than in patients with significant or severe disturbance of consciousness (GCS scores 9–11 or ≤8), respectively. Cases that were ultimately fatal were most frequently associated with low GCS scores at hospital admission. However, some of the deaths occurred in patients with initially high GCS scores at the time of reporting and few to no neurological deficits. For these patients, massive intracranial bleeding and brain death was reported in the context of the deterioration of the patient’s condition or cause of death.

To explore the relationship between clinical outcomes and the degree of thrombocytopenia, we examined GOS-E scores in 44 patients together with the lowest available thrombocyte counts (Fig. 1B). Indeed, platelet count nadirs were correlated with patient outcomes (Spearman correlation coefficient, *r* = 0.73, *p* < 0.001). Low platelet counts were observed in fatal cases (GOS-E 1) with a mean count of 17,000 platelets/μL). Likewise, patients with better neurological outcomes (GOS-E scores of 5–6 and 7–8) presented with mean counts of 61,000 thrombocytes/μL.

However, the course of the disease was not always predictable and showed significant individual variability. In one case, a male patient presented with headache, thrombocytopenia, and increased d-dimer levels with no signs of CVST on diagnostic imaging at 10 days post-vaccination. One week later, a second MRI performed to address his deteriorating neurological status revealed fulminant CVST. The patient was transferred to a specialized stroke unit, where he sustained an intracranial hemorrhage and died a few days later, nearly one month following vaccination.

By contrast, good recoveries were reported for several of the more severe cases, including a patient with CVST who was treated with anticoagulation and IVIG but exhibited neurological deterioration on the following day with evidence of cerebral hemorrhage requiring an emergency mechanical thrombectomy. The patient’s condition went on to stabilize and platelet numbers ultimately returned to normal levels.

## Discussion/conclusion

We provide data on the outcome of VITT cases with CVST upon vaccination with Vaxzevria^®^ and found that the recovery of patients from CVST was very heterogeneous. While some patients exhibited good recoveries, others developed severe disabilities and major long-term complications.

Nearly half of the patients in our follow-up investigation had either a fatal outcome or severe neurological disabilities 3–6 months after hospital admission. Of the 30 individuals who survived CVST, 12 patients were no longer able to work (GOS-E scores of 2–5); nine patients had only limited ability to work (GOS-E scores of 6 and 7). Most of the patients exhibited persistent mental disorders (disruption of social activities and their social lives). At the time that these data were collected, only 9 of the 44 patients in our investigation were able to resume their previous life with no restrictions (GOS-E score of 8). We presume that the higher number of females in our investigation is attributed to the vaccine recommendations valid at that time (please refer to Supplementary Information for details).

We observed a mortality rate of 32%, which is comparable to similar investigations after using the ChAdOx1 nCoV-19 vaccine^3,5,10^; this rate appears to be higher than that reported for cases of CVST with different pathogenesis^11^. The causes for the high fatality rate in our population remain speculative. The small sample size and retrospective design preclude any approaches involving rigorous correlations. Since CVST is a non-routine diagnosis in general practice (and even more so in response to vaccination), delays in reaching a correct diagnosis may have contributed to fatalities and poor outcomes in some of these cases.

As anticipated, initial low GCS scores were associated with poor outcomes. By contrast, GCS scores >10 were typically associated with improved neurological outcomes. We found that low platelet counts were also related to poor patient outcomes. Twenty-one of the 28 patients with low platelet counts (<50,000/µL) showed not only cerebral venous thrombosis but also cerebral bleeding, a finding that further complicated the course of the disease.

Our investigation exhibits several limitations, some of which are inherent to spontaneous adverse reaction reporting: The small sample size, loss of follow-up (10.2%), missing information, and outcome assessments after varying periods. Overall, this restricts the conclusions that can be drawn from the investigation and do not allow any interpretation with regards to the efficacy of specific treatment regimes, nor can our findings be expanded to thromboses in other locations. Moreover, the fact that no information on new thrombosis, major bleeding, and recurrence of thrombocytopenia was collected is another limitation of this investigation.

Collectively, our findings highlight the importance of paying attention to early signs of increased intracranial pressure and the onset of thrombocytopenia in patients with a recent history of vaccination. In this case, early imaging and close patient monitoring is required. Timely diagnosis is critical to effective management and mitigation of the potential adverse outcomes of these vaccine recipients.

While the frequency of serious VITT with CVST after vaccination with the ChAdOx1 nCoV-19 vaccine remains low (e.g., estimated incidences of VITT are in the range of 1:50,000 to 1:150,000^5,12^) this risk associated with this vaccine and potentially with other adenoviral vector vaccines as well^13^ must be taken seriously. For a national vaccination strategy, the availability and risks of other vaccines should be taken into consideration and carefully evaluated. Based on the proposed mechanism of this ADR, recommendations for diagnosis and management are being developed to reduce mortality and improve outcomes^8,9,14^. Potential implications of possible risk factors on the reaction or outcome were not apparent in our investigation. This leads to a lack of predictive factors defining particularly vulnerable populations and thereby precludes risk-minimizing measures.

## Methods

### Data collection

As described in Thiele et al.^15^, relevant individual case safety reports of patients with thrombosis (*n* = 854) and/or thrombocytopenia (*n* = 224), of whom 106 cases met the interim case definition of the Brighton Collaboration^4^, were identified after Vaxzevria^®^ vaccination (reported from 01-Feb-2021 until 21-May-2021) by a PEI database query. Experts from the PEI manually reviewed case reports of TTS. A subgroup of these reports was evaluated for the current investigation that included all cases of CVST confirmed by medical imaging (Brighton Collaboration Definition levels 1 and 2^4^). We initially identified 54 cases with dates of vaccination that included 13-Feb-2021 through 07-May-2021. We eliminated four duplicates and one case in which follow-up revealed no CVST. The remaining 49 cases were followed up for the current neurological status of the patients (Suppl. Figure 1). Data on new adverse events during follow-up was not collected. In case more than one platelet count per individual was reported (e.g., platelet counts on different days during hospitalization), the lowest value was used regardless of its timing relative to surgical intervention (Fig. 1B). All of the patients showed an onset of symptoms 5–30 days after vaccination against SARS-CoV-2 as well as the presence of thrombosis (CVST) and thrombocytopenia (platelet count <150,000 per cubic millimeter). For 34 patients d-dimer levels >0.5 mg/L and for 41 patients positive anti-PF4 antibodies on ELISA were reported. Overall, in regards to the Pavord criteria^5^, 29 patients meet the criteria for definite VITT, 17 patients fulfill the criteria for probable VITT and 3 patients meet the criteria for possible VITT.

### Glasgow Coma Scale (GCS) scores

Individual GCS scores were reported by phone or electronically via a questionnaire or medical report by the treating physician of the hospital to which the patient was initially admitted. If a GCS score was not reported, an expert determined a score based on the patient’s medical report. *N* = 23 GCS scores were reported and *n* = 20 GCS scores were determined based on the patient’s medical report. In cases in which more than one GCS score was documented, the lower value was chosen.

### Extended Glasgow Outcome Scale

Scientific assessors of the PEI contacted the initially treating physicians or the institutions providing further treatment (e.g., rehabilitation centers or general practitioners) by phone or via an electronic questionnaire. Physicians were asked for information on the current neurological status of each patient using the extended GOS-E. Results were collected in written form. In four cases, the patient’s status could not be assigned to a single GOS-E score (7–8, 5–6, 7–8, and 4–5) and the higher category was chosen for analysis. Data were evaluated using the assessment of two experts.

### Statistical analysis

With respect to qualitative variables, relative frequencies were calculated, and regarding quantitative variables mean, median as well as 25 and 75% percentiles. Descriptive statistics were performed using Microsoft Excel (2016). To test for correlation between the overall outcome and GCS at admission as well as the overall outcome and platelet count nadirs, Spearman correlation coefficients were calculated with *α* = 0.05. The correlation analysis was conducted using SAS, version 9.4 (SAS Institute, Cary, NC/USA).

### Ethics

The follow-up of vaccination complication reports in a pseudonymised form is covered by the legal mandate of the Paul-Ehrlich-Institut according to the German Medicinal Products Act (AMG §62) and the Infectious Protection Act. The responsible Ethics committee (Ethikkommission bei der Landesärztekammer Hessen) was consulted and accepted the follow-up procedure as described herein. In agreement with the Ethics committee, no written informed consent by the participants was necessary.

### Reporting summary

Further information on research design is available in the Nature Research Reporting Summary linked to this article.

## Supplementary information

Supplementary Information

Reporting Summary

## Data Availability

The data set analyzed in the current investigation is available from the corresponding author upon reasonable request.
