# Supplementary material for: Long-term outcome of patients with vaccine-induced immune thrombotic thrombocytopenia and cerebral venous sinus thrombosis
Source: NPJ Vaccines. 2022 Jul 5;7:76. doi: 10.1038/s41541-022-00491-z (PMC9255460; doi:10.1038/s41541-022-00491-z)
Supplement: Supplementary file 1 — Supplementary Information [file 41541_2022_491_MOESM1_ESM.pdf]

Supplementary Information

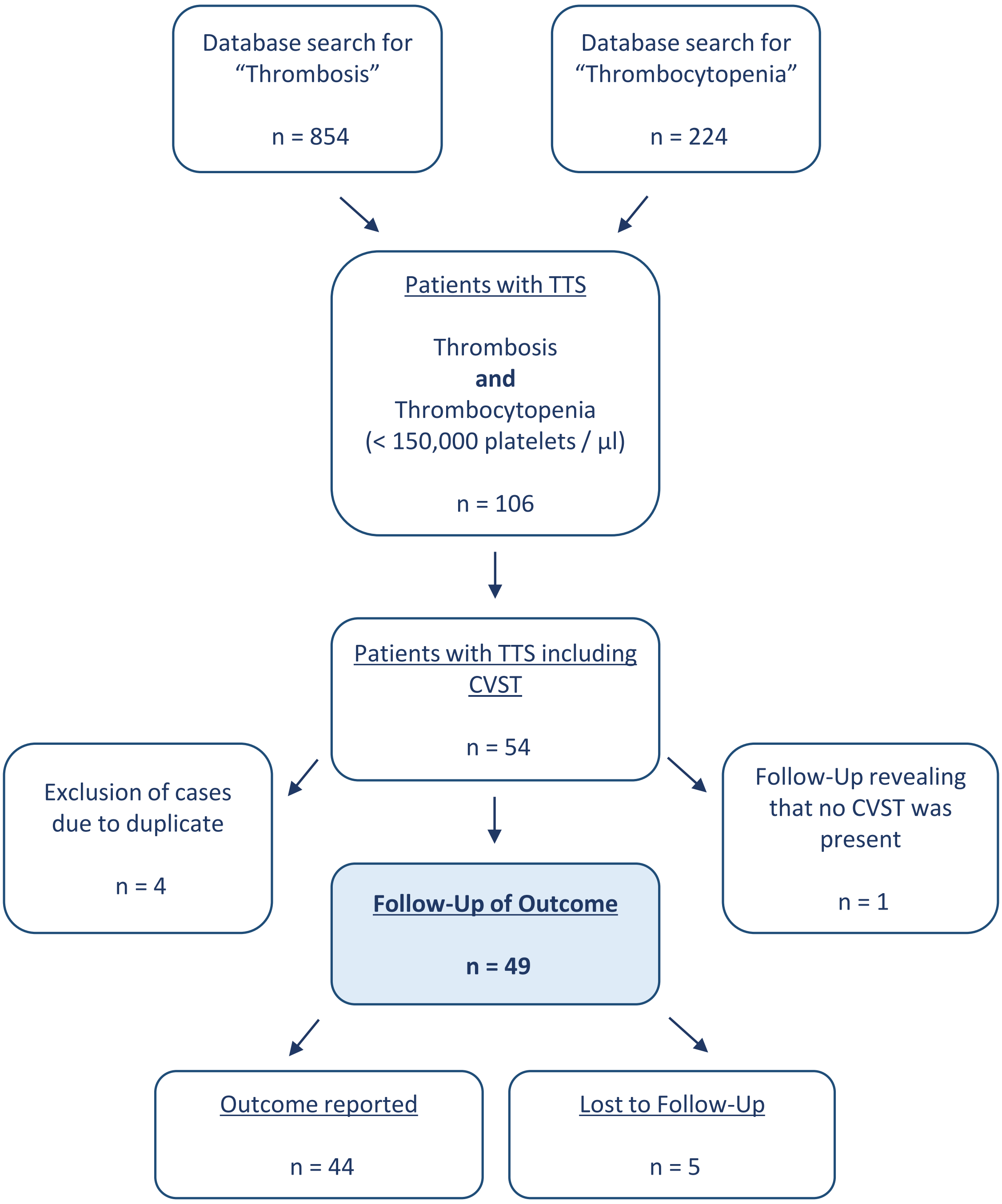

Supplementary Figure 1: Notified cases of thrombosis, thrombocytopenia, TTS and TTS including CVST in Germany (01-Feb-2021 – 21-May-2021) after ChAdOx1 nCoV-19 COVID-19 (Vaxzevria®) vaccination.

Supplementary Table 1: Characteristics of VITT patients (n=49) with CVST

	Number of patients (total n=49)
Gender	
Female	38
Male	11
Age Category [years]	
18-39	21
40-59	15
60+	13
Mean (Min-Max)	45.1 (19-73)
Period Vaccination – Date of admission to hospital [days]	
1 week (1-7 days)	7
2 weeks (8-14 days)	34
3 weeks (15-21 days)	8
Mean (Min-Max)	11.3 (5-19)

Supplementary Table 2: Risk factors of VITT patients (n=49; 38 females, 11 males) with CVST

	Number of patients with no information on risk factor available	Number of patients with information on risk factor available	Number of patients with reported risk factor
Smoking	17	32	2
Contraceptives	14	24	9
BMI >35 kg/m^2	22	27	2
History of thrombosis	48	1	1

Supplementary Note

Vaccination recommendations in Germany

In accordance with the recommendation of the German Standing Committee on Vaccination (STIKO) from February 2021 to the end of March 2021[(1)], the ChAdOX1 nCoV-19 vaccine Vaxzevria® was administered to younger people (<65 years) who fulfilled high priority criteria (medical staff and nursing personnel, school teachers, informal caregivers). When thromboses at unusual sites including cerebral venous sinus thrombosis (CVST) in combination with thrombocytopenia emerged as an adverse reaction in patients vaccinated with the ChAdOx1 nCoV-19 vaccine, the STIKO revoked its original recommendation to vaccinate patients under 65 on 30-Mar-2021 and instead recommended vaccinating those ≥60 years with the ChAdOx1 nCoV-19 vaccine. Since women are overrepresented in many of the high priority professions (medical staff, (primary school) teachers, caregivers etc.), more females than males were vaccinated with the ChAdOx1 nCoV-19 vaccine in February and March. In this period, the majority of the patients with VITT and CVST included in this investigation were vaccinated.

(1): Vygen-Bonnet S *et al.* Beschluss der STIKO zur 2. Aktualisierung der COVID-19-Impfempfehlung und die dazugehörige wissenschaftliche Begründung Epid Bull 2021;5:3 -79 | DOI 10.25646/7820.3
