# Supplementary material for: Long-term outcome of patients with vaccine-induced immune thrombotic thrombocytopenia and cerebral venous sinus thrombosis
Source: NPJ Vaccines. 2022 Jul 5;7:76. doi: 10.1038/s41541-022-00491-z (PMC9255460; doi:10.1038/s41541-022-00491-z)
Supplement: Supplementary file 2 — Reporting Summary [file 41541_2022_491_MOESM2_ESM.pdf]

nature portfolio

Corresponding author(s): Prof. Dr. Markus Funk

Last updated by author(s): Mar 22, 2022

# Reporting Summary

Nature Portfolio wishes to improve the reproducibility of the work that we publish. This form provides structure for consistency and transparency in reporting. For further information on Nature Portfolio policies, see our Editorial Policies and the Editorial Policy Checklist.

## Statistics

For all statistical analyses, confirm that the following items are present in the figure legend, table legend, main text, or Methods section.

| n/a | Confirmed | |
|---|---|---|
| ☐ | ☒ | The exact sample size (*n*) for each experimental group/condition, given as a discrete number and unit of measurement |
| ☒ | ☐ | A statement on whether measurements were taken from distinct samples or whether the same sample was measured repeatedly |
| ☐ | ☒ | The statistical test(s) used AND whether they are one- or two-sided<br>*Only common tests should be described solely by name; describe more complex techniques in the Methods section.* |
| ☒ | ☐ | A description of all covariates tested |
| ☒ | ☐ | A description of any assumptions or corrections, such as tests of normality and adjustment for multiple comparisons |
| ☐ | ☒ | A full description of the statistical parameters including central tendency (e.g. means) or other basic estimates (e.g. regression coefficient) AND variation (e.g. standard deviation) or associated estimates of uncertainty (e.g. confidence intervals) |
| ☐ | ☒ | For null hypothesis testing, the test statistic (e.g. *F*, *t*, *r*) with confidence intervals, effect sizes, degrees of freedom and *P* value noted<br>*Give P values as exact values whenever suitable.* |
| ☒ | ☐ | For Bayesian analysis, information on the choice of priors and Markov chain Monte Carlo settings |
| ☒ | ☐ | For hierarchical and complex designs, identification of the appropriate level for tests and full reporting of outcomes |
| ☐ | ☒ | Estimates of effect sizes (e.g. Cohen's *d*, Pearson's *r*), indicating how they were calculated |

*Our web collection on statistics for biologists contains articles on many of the points above.*

## Software and code

Policy information about availability of computer code

| | |
|---|---|
| Data collection | MS Office (Excel, Word; 2016) |
| Data analysis | MS Office (Excel, PPT 2016), SAS (Version 9.4), SAS Institute, Cary, NC/USA |

For manuscripts utilizing custom algorithms or software that are central to the research but not yet described in published literature, software must be made available to editors and reviewers. We strongly encourage code deposition in a community repository (e.g. GitHub). See the Nature Portfolio guidelines for submitting code & software for further information.

## Data

Policy information about availability of data

All manuscripts must include a data availability statement. This statement should provide the following information, where applicable:

- Accession codes, unique identifiers, or web links for publicly available datasets
- A description of any restrictions on data availability
- For clinical datasets or third party data, please ensure that the statement adheres to our policy

The dataset analyzed in the current investigation is available from the corresponding author upon reasonable request.

# Field-specific reporting

Please select the one below that is the best fit for your research. If you are not sure, read the appropriate sections before making your selection.

☒ Life sciences ☐ Behavioural & social sciences ☐ Ecological, evolutionary & environmental sciences

For a reference copy of the document with all sections, see nature.com/documents/nr-reporting-summary-flat.pdf

# Life sciences study design

All studies must disclose on these points even when the disclosure is negative.

| | |
|---|---|
| Sample size | No sample-size calculation was performed. Subjects were followed up from initial reports received by the Paul-Ehrlich-Institut in the frame of spontaneous serious adverse reaction reporting. All cases meeting certain criteria were followed up irrespective of sample size calculation. |
| Data exclusions | No data was actively excluded. During our investigation 5 cases were lost to follow-up. |
| Replication | Since our analysis includes data from individual patient's outcome, no replication was performed. |
| Randomization | This was not relevant to our study since we describe patient's outcome after CVST according to a predefined GOS-E scale according to which they were classified. |
| Blinding | Blinding was not relevant nor possible in our study since subjects were followed up from initial reports received by the Paul-Ehrlich-Institut in the frame of spontaneous serious adverse reaction reporting. Patients were chosen by predefined criteria according to the Brighton Collaboration (please refer to the mansuscript for details). |

# Reporting for specific materials, systems and methods

We require information from authors about some types of materials, experimental systems and methods used in many studies. Here, indicate whether each material, system or method listed is relevant to your study. If you are not sure if a list item applies to your research, read the appropriate section before selecting a response.

## Materials & experimental systems

| n/a | Involved in the study | |
|---|---|---|
| ☒ | ☐ | Antibodies |
| ☒ | ☐ | Eukaryotic cell lines |
| ☒ | ☐ | Palaeontology and archaeology |
| ☒ | ☐ | Animals and other organisms |
| ☐ | ☒ | Human research participants |
| ☒ | ☐ | Clinical data |
| ☒ | ☐ | Dual use research of concern |

## Methods

| n/a | Involved in the study | |
|---|---|---|
| ☒ | ☐ | ChIP-seq |
| ☒ | ☐ | Flow cytometry |
| ☒ | ☐ | MRI-based neuroimaging |

## Human research participants

Policy information about studies involving human research participants

| | |
|---|---|
| Population characteristics | Forty-nine patient cases were evaluated in this investigation. Of these, 38 patients were female and 11 were male, which likely reflects vaccination recommendations at that time (please refer to the Supplementary Information for details) (Suppl. Table 1). The mean age was 45 years (range 19 – 73 years), and most patients (n = 34) were admitted to the hospital during week 2 after vaccination. Seven patients were admitted to hospital during week 1 after vaccination and 8 patients were hospitalized during week 3 upon vaccination (overall mean 11 days, range 5 – 19 days). Spontaneous reporting details on ADR management are not provided on a routine basis, but medical reports were requested for this particular reaction. Twenty-three of these patients underwent a surgical procedure, including 15 patients who required craniectomies to reduce intracranial pressure (6 survived). Catheter-assisted thrombectomy was performed in 9 cases (including 2 craniotomies with thrombectomy). One patient required implantation of a ventricular drainage device due to cerebrospinal fluid circulation disorder. For 22 patients intravenous immune globulin (IVIG) treatment was reported. Half of these patients (n = 11) additionally underwent a surgical procedure. In 35 patients, treatment with low-molecular-weight heparin, non-heparin anticoagulants, or other medications such as corticosteroids and/or platelet transfusions was reported. In this context, it has to be noted that at the time when most reports were received, no guidelines for VITT treatment were available or they were about to be generated8. Concerning possible risk factors associated with CVST (Suppl. Table 2), two of the patients were smokers (smoking status was reported for 32 patients), and 9 patients used hormonal contraceptives (information on contraceptive intake was available for 24/38 female patients). Weight and height were known for 27 patients. However, only two patients presented with an abnormally high BMI>35 kg/m2. History of thrombosis was described in 1 patient, and only 1 patient showed two or more risk factors. |

| | |
| --- | --- |
| Recruitment | Subjects were not actively recruited, but were followed up from initial reports received by the Paul-Ehrlich-Institut in the frame of spontaneous serious adverse reaction reporting. Subjects were followed up anonymously. |
| Ethics oversight | The follow-up of vaccination complication reports in a pseudonymised form is covered by the legal mandate of the Paul-Ehrlich-Institut according to the German Medicinal Products Act (AMG §62) and the Infectious Protection Act. The responsible Ethics committee (Ethikkommission bei der Landesärztekammer Hessen) was consulted and accepted the follow-up procedure as described herein. |

Note that full information on the approval of the study protocol must also be provided in the manuscript.
